# Hemodynamic targets during therapeutic hypothermia after cardiac arrest: a prospective observational study

**DOI:** 10.1186/cc14506

**Published:** 2015-03-16

**Authors:** K Ameloot, I Meex, C Genbrugge, W Boer, F Jans, B Ferdinande, W Mullens, M Dupont, C Dedeyne, J Dens

**Affiliations:** 1 ZOL Genk, Belgium

## Introduction

In analogy with sepsis, current postcardiac arrest (post-CA) guidelines recommend to target mean arterial pressure (MAP) above 65 mmHg and SVO_2_ above 70%. This is unsupported by mortality or cerebral perfusion data. The aim of this study was to explore the associations between MAP, SVO_2_, cerebral oxygenation and survival.

## Methods

A prospective, observational study during therapeutic hypothermia (24 hours -33°C) in 82 post-CA patients monitored with near-infrared spectroscopy.

## Results

Forty-three patients (52%) survived in CPC 1 to 2 until 180 days post CA. The mean MAP range associated with maximal survival was 76 to 86 mmHg (OR = 2.63, 95% CI (1.01; 6.88), *P *= 0.04). The mean SVO_2_ range associated with maximal survival was 67 to 72% (OR = 8.23, 95% CI (2.07; 32.68), *P *= 0.001). In two separate multivariate models, a mean MAP (OR = 3.88, 95% CI (1.22; 12.33), *P *= 0.02) and a mean SVO_2_ (OR = 8.79, 95% CI (1.69; 18.36), *P *= 0.01) in the optimal range persisted as independently associated with increased survival after correction for presence of early bystander CPR and presenting shockable rhythm. Based on more than 1,625,000 data points, we found a strong linear relation between SVO_2_ (range 40 to 90%) and average cerebral saturation (*R*^2 ^= 0.86) and between MAP and average cerebral saturation for MAPs between 40 and 87 mmHg (*R*^2 ^= 0.70). Based on our hemodynamic model, the optimal MAP and SVO_2_ were determined to be 87 mmHg and 72%.

## Conclusion

The optimal SVO_2_ (72%) and MAP (87 mmHg) derived from our hemodynamic model matched with the observed SVO_2_ (67 to 72%) and MAP (76 to 86 mmHg) associated with maximal survival. Prospective interventional studies to reach or maintain these targets are needed to confirm these findings.

